# Media capture strategies in new authoritarian states: the case of Turkey

**DOI:** 10.1007/s11616-020-00600-9

**Published:** 2020-09-16

**Authors:** Gülçin Balamir Coşkun

**Affiliations:** Kultur‑, Sozial- und Bildungswissenschaftliche Fakultät, Institut für Sozialwissenschaften, Humboldt-Universität zu Berlin, Unter den Linden 6, 10099 Berlin, Germany

**Keywords:** Media capture, Turkey, AKP, Ownership, Financial sanctions, Criminalizing journalists

## Abstract

This article focuses on the forced transformation of the mass media as an institution in new authoritarian states. It aims to understand the methods used by theses states to control and manipulate the flux of news through the mass media. Turkey’s media system has been chosen as a case study because the recent political developments in the country offer worrisome und devastating examples. This article aims to answer to the following question: How can we classify methods and strategies used by the AKP government to capture the media in Turkey? Why and how do the methods used by the AKP government differ from those applied by previous governments? To answer to these questions, the article draws on media capture as a framework of analysis. It argues that the AKP captured the media by using new strategies which can be divided into three overlapping and interconnected categories: capture by creating its own private media, capture through financial sanctions, and capture by intimidating and criminalizing journalists.

## Introduction

The 21st century started with the rise of a new kind of authoritarian regime that hides behind institutional façades of representative democracy in different parts of the world, from Latin America to Europe and Asia. This new phenomenon called into doubt previous optimistic analyses of the worldwide transition to democracy and pushed political scientists to think about the relationship between democratic institutions and authoritarian transformation. Some scholars prefer to define these new kinds of regime as authoritarian electoral regimes that “neither practice democracy nor resort regularly to naked repression” (Schedler 2002, p. 36) while others name them “illiberal democracies” (Buzogány 2017; Plattner 2019; Zakaria 1997) or “competitive authoritarianism” (Levitsky and Way 2002). Although there is no agreement on how to characterize today’s undemocratic regimes, there is “an emerging consensus on one important insight: the process of autocratization seems to have changed” (Lührmann and Lindberg 2019, p. 1097). New authoritarian regimes do not reject democracy itself, they reduce it to majoritarianism. Although they retain the electoral system, they tend to change the rules of the game and to transform or destroy political institutions such as the legislature, independent courts, elections, political parties, the media, civil society, and subnational units (cf. Schedler 2013, p. 328). Each of these institutions plays an important role in the functioning of democratic system. Analyzing and clarifying the methods used to redesign these institutions helps us to grasp the latent authoritarian engineering behind this process and to develop alternative ways of fighting against it.

This article focuses on the forced transformation in newly authoritarian states of the mass media as an institution. It aims to understand the methods used by these states to control and manipulate the flux of news through the mass media. Turkey’s media system has been chosen as a case study, because the recent political developments in the country, characterized as “competitive authoritarianism” (Esen and Gümüşçü 2016; Özbudun 2015) or “electoral authoritarianism” (Coskun and Kölemen 2020; Kaya 2015), show that the authoritarian turn has been conceived under a façade of democracy. Against this backdrop, this article aims to answer to the following question: How can we classify methods and strategies used by the AKP (Adalet ve Kalkınma Partisi—Justice and Development Party) government to capture the media in Turkey? Why and how do the methods used by the AKP government differ from those applied by previous governments?

To answer to these questions, the article draws on media capture as a framework of analysis, which will be described in Sect. 2. Sect. 3 focuses on Turkey’s media landscape from the time of the military coup d’état in 1980 to the 2000s to understand the foundation that permitted the AKP government to develop its policies to capture the media without hindrance. Then, in the fourth section, I analyze and classify the methods and strategies used by the AKP government. This analysis is based on a review of the secondary literature on AKP’s media policies as well as on a media review and 22 interviews I conducted with journalists. In conclusion, I explain how the AKP’s methods differ from the strategies of earlier governments.

## Media capture as a framework of analysis

The democratic process of voting presupposes that citizens have sufficient knowledge about the government’s policies and performance as well as the opposition’s critics and promises to choose between them. As democratic governments have to legitimize themselves through elections, public opinion about their performance is important. The public opinion is influenced by family inherence and personal experience as well as by the public knowledge on political issues framed and transmitted via media (cf. Forman 1991, p. 109). This public knowledge constitutes an important source for citizens to check governments’ policies and to decide among different political actors. The decisions, actions, and reactions by different political actors on socioeconomic issues “are selected and shaped by mass-media professionals” (Habermas 2006, p. 415) and thus become accessible to the public. For this reason, some scholars describe the news as a main agent in the “construction of public knowledge” (Schudson 2002). In brief, mass media play an indispensable role in the formation of this public knowledge.

Having understood the important role of the media in politics, new authoritarian regimes have developed new strategies to influence the formation of public knowledge by the media. Instead of establishing direct state control over the media, they tolerate or encourage the private media. However, they prevent them from producing critical content using “diverse measures such as national security prosecutions, punitive tax audits, manipulation of government advertising, and seemingly reasonable content restrictions” (Simon 2015, p. 33). To explain the new relation between the media and the new authoritarians, scholars have drawn on economic theories of capture (cf. Besley and Prat 2006; Corneo 2006; Petrova 2008).

The “regulatory capture” or “capture theory,” associated with the work by economist George Stigler, *The Theory of Economic Regulation* (1971), draws attention to the fact that regulatory agencies may come to be dominated by the interests they regulate and not by the public interest. Stigler’s work helps us to understand the main problems not only in the governmental regulations but also in the other sectors that are supposed to fulfill a regulatory or monitoring function in democracies (cf. Carrigan and Coglianese 2016, p. 1). The usefulness of the broad analytical framework that the term “capture” offers soon became obvious to scholars working on power relations and the media.

According to the most common definition, the term media capture points to “a situation in which the media have not succeeded in becoming autonomous in manifesting a will of their own, nor able to exercise their main function, notably of informing people. Instead, they have persisted in an intermediate state, with vested interests, and not just the government, using them for other purposes” (Mungiu-Pippidi 2013, p. 41). Based on this definition Stiglitz proposes “a taxonomy of media capture based on four broad, and somewhat overlapping, sections: (a) ownership, (b) financial incentives, (c) censorship, and (d) cognitive capture” (Stiglitz 2017, p. 11–14). While capture through ownership refers to the purchase of mass media institutions by wealthy individuals and corporations and to the creation of interest relations between these owners and the political elites, capture through financial incentives is related to the incentives of advertising and to the relationship between advertisers and media outlets. In the third category of his taxonomy Stiglitz talks very briefly about the relationship between censorship and capture and focuses on journalists’ self-censorship due to their oppression by the government as well as on the commercial interests of media corporations. Lastly, Stiglitz discusses cognitive capture, which he defines as “the most interesting aspect of capture—the most subtle, the hardest to prove”. This refers to a situation where journalists lose their critical and investigative reflexes and start to transmit the dominant point of view.

Although Stiglitz’s taxonomy focuses mainly on media capture by economic elites, recent developments in new authoritarian states, or and perhaps also in so-called democratic countries like the USA, Italy or the UK, necessitate a more detailed analysis of the ways of media capture by governments. There are growing numbers of studies of media capture by governments, which aim not merely to censor but to manipulate. Mungiu-Pippidi (2008, p. 73) argues that some governments in eastern Europe are unable or unwilling to impose direct control over the media, but prefer to capture them by state subsidies, the preferential distribution of state advertising or by handing out financial favors and penalties. In exchange for these favors or penalties, they aim to get media coverage or framing favorable to their political agenda. The concentration of the media and existing clientelist relationships has brought about media capture by economic elites as well as political leaders (cf. Márquez-Ramírez and Guerrero 2017, p. 54) and these authors explain to what extent these captured media promote the private interests of media executives and politicians over the public good.

A number of recent studies of the Turkish media system also use the media capture framework in their analyses of the legal and financial pressures on media organizations and journalists. Finkel (2015) surveys the historical background of media concentration and explains how it has facilitated media capture by the AKP government. Yanatma’s research “examines advertising as a tool of government to control the media, particularly print media in a country where politics and journalism have long been intertwined in an often combustible mixture” (Yanatma 2016, p. 6). Yeşil’s article (2018) focuses on the disciplinary strategies deployed by the AKP and reveals the connection between its capture of the media and the previous authoritarian neoliberal order. Yet, the number of studies using capture theory to analyze media system in Turkey is still limited. This articles aims to fill this gap as well as to classify capture strategies used by the AKP to create the basis for comparative research among new authoritarian states. However, before analyzing these strategies, I offer a brief overview of crucial 20 years that paved the way for the rise of the AKP and the transformation of the media system in the 2000s.

## Key characteristics of the media landscape in Turkey after the 1980 military coup d’état

After the 1980 coup d’état a new Constitution was designed under the tutelage of Turkish military forces and entered into force in 1982. Article 28 of the new Constitution regulates the freedom of press and starts by saying that “The press is free, and shall not be censored”. However, the following lines of the same article define in detail the conditions under which the state can ban and take the measures necessary to stop the distribution of newspapers and periodicals. The main reason for this ban, according to Article 28, is to protect the internal and external security of the state, which can be interpreted very broadly. Additionally, different articles of the penal code (such as Articles 132, 137, 161 and 162) bear directly or indirectly upon the press and were vaguely defined so that the judiciary could use them against journalists when the “state interest” was in danger (cf. Waldman and Çalışkan 2019, p. 386).

This illiberal constitutional and legal order was not the only characteristic of the journalistic environment in Turkey post coup d’état. Neoliberal winds had knocked on Turkey’s door. One of the reasons for the 1980 coup d’état was to facilitate the application of a new economic policy aiming to integrate and adapt Turkish economy to the global capitalist system (cf. Gürsel 1995). The framework of this economic policy was set by decisions made on January 24, 9 months before the coup d’état. One of these decisions was directly related to the media sector and stipulated the end of the state subventions previously granted to newsprint. This decision slowly made all newspapers financially dependent on advertisement revenue. This neoliberal transformation of economic policies created a completely new environment where media owners with a journalistic family background could not easily find ways to afford the cost of paper and to get sufficient advertisements to finance their newspapers (cf. Sözeri and Güney 2011, p. 39). There were two options for these traditional owners: either to invest in areas outside the media or to sell their family business to new entrepreneurs with backgrounds in banking or construction (cf. Kaya 2016, pp. 261–262). As a result, the ownership profile of the media slowly changed. By the second half of the 1990s only a limited number of conglomerations, chiefly those owned by Doğan, Bilgin and Doğuş Holdings, had started to dominate the media market. Their main aim was to “obtain political patronage and support for their other business ventures” (Kaya and Çakmur 2010, p. 530). The weak coalition governments and economic instability in the 1990s obviously helped these new media conglomerates to pull the strings of political leaders and to steer the game in the direction that they desired. Later, when Erdoğan decided to get rid of these big media holdings, he used this corrupted relationship between media owners and previous political leaders as a card against them.

The third characteristic of the media landscape in the 1980s and 1990s was absolute censorship on the Kurdish issue and high levels of violence against Kurdish journalists or defenders of human rights. This censorship took a form deliberately ignoring the Kurds at the beginning of 1980s. While the military rule imposed restrictions on Kurdish identity such as changing the names of Kurdish cities or prohibiting the use of the Kurdish language in public, the media preferred not to cover topics related to these steps. On the very rare occasions when they did treat on topics related to the Kurds or Kurdish regions, they avoided using use the word “Kurd”. In fact, it was a reflection of Turkish state discourse (cf. Yeğen 1999) imposed and controlled by the military rule. The legal framework allowed for lengthy prison sentences to journalists who did not accept to stay within the limits of the state discourse. Somer’s research, for shows, reveals that “in 1984 and 1985, the mainstream Turkish daily *Hürriyet *published only 25 articles that were fully or partially related to the country’s ethnic Kurds. Only 3 of these 25 articles used the word Kurd in reference to a person, group, concept, or place” (Somer 2005, p. 591). This intentional ignorance by the mainstream media in the beginning of the 1980s was later transformed into a framework of enmity directed by the military when the armed conflict between the Partiya Karkeren Kurdistane—Kurdistan Workers’ Party (PKK) and the Turkish army intensified at the beginning of the 1990s. At the same time, various human rights organizations had prepared reports on the murder, torture, depopulation and extrajudicial killings targeting firstly Kurds, but also leftist human rights defenders, advocates, and journalists. However, mainstream newspapers, voluntarily or involuntarily, turned a blind eye to all human rights violations (cf. Waldman and Çalışkan 2019, p. 389). Some journalists working for left-wing or pro-Kurdish newspapers were killed by “unknown people” and the Turkish government made no serious effort to investigate these killings. When Demirel, then prime minister, was asked about the imprisonment or killings of journalists, he answered without shame: “Those killed were not real journalists. They were militants in the guise of journalists. They kill each other.” (HRW 1993, p. 18) Labeling journalists who preferred to practice their profession outside the state-drawn framework as “terrorists”, which Erdoğan often did, has its roots in the recent history of journalism in Turkey.

The fourth characteristic of the media landscape is the weakening of union rights and the increasing oppression on journalists who were members of unions. This de-unionization was not specific to the media sector. In fact, it was directly related to the scything of left-wing organizations active in the 1970s as well as to the neoliberal economic policies of Turkish governments in the 1980s. The cooperation of the conglomerates dominating the media sector together with the government facilitated this de-unionization (cf. Özsever 2004, p. 159). In practice, union employees started to be dismissed because they were union members and the monopoly structure of the sector did not permit them to find employment in other media enterprises. At this time of the crisis, the failure of the unions to offer support to the dismissed and threatened journalists also played a role in journalists’ unwillingness to become union members (cf. Sözeri and Güney 2011, p. 32).

In brief, when the AKP came to power in 2002, journalists were working without any constitutionally or legally guaranteed freedom. Additionally, some political questions such as the Kurdish issue were considered taboo and could only be treated in the way defined by the state bureaucracy and the military. The neoliberal economic transformation in Turkey also shaped journalists’ working conditions and rights. The clientelistic relationship and the interaction between media patrons and political leaders were accepted as natural.

## Media capture strategies in the AKP era

As this brief snapshot of historical media-state relations shows, media freedom has never fully existed in Turkey. I argue that when the AKP came to power they used the previous laws limiting the freedom of the media, established clientelistic relations between the media patrons and political leaders, and provided weak protection to media professionals as a starting point. Consecutive victories in elections fueled their desire and capacity to capture the whole state apparatus. In this process, controlling, using and manipulating information according to their interests played a crucial role. The AKP government did not challenge the neoliberal media and did not try to nationalize private media outlets. They captured the media using new strategies. Instead of giving a timeline of AKP’s media policies, this section aims to classify these strategies to allow further comparative research. The classification is based on Stiglitz’s taxonomy, but calibrates it according to practices in a new authoritarian state, namely Turkey.

We can classify the AKP’s methods in three categories: capture by creating its own private media, capture through financial sanctions, and capture by intimidating and criminalizing journalists.

### Capture by creating its own private media

At the end of 1990s four major holding companies were dominant in the mainstream media sector in Turkey: Doğan, Bilgin, Uzan, and Çukurova. According to the report presented to the Media Inquiry Committee of the Turkish Grand National Assembly in 2002, 84% of the daily newspapers sold in Turkey belonged to the Doğan, Bilgin or Çukurova group (cf. Özsever 2004, p. 125). The 2001 economic crisis brought about a crash, especially in the banking sector, which directly influenced the Bilgin and Uzan groups. The Tasarruf Mevduatı Sigorta Fonu—the Savings Deposit Insurance Fund (TMSF) expropriated their media assets, including several newspapers, television and radio outlets. While these two important groups were excluded from the media sector, two new big actors joined the club of media patron, namely Doğuş and Ciner (cf. Yeşil 2018, p. 245). Although there were important differences and even some conflicting interests among these media patrons (cf. Özsever 2004, p. 122), they had at least two areas in common. Firstly, as supporters of neoliberal policies, they were opposed to any kind of journalist organization. Secondly, they were not members of the AKP’s close circle.

During its first term the AKP government tried to influence the mainstream media dominated by these groups using a carrot and stick strategy, like previous governments. At the same time, some other media groups such as Ihlas, Albayrak, and Gülen^1^’s newspapers and TV channels with Islamist backgrounds were becoming richer (cf. Özsever 2004, p. 120). However, the circulation numbers and ratings of these partisan media group outlets were limited. The AKP realized soon that they had to develop a new ownership profile to have more direct control over the management of newspapers and TV channels to influence the “construction of public knowledge”. Businessmen from Erdoğan’s inner circle who had started to prosper under the AKP government had to be encouraged and, if necessary, pushed to buy mainstream media outlets. To create a partisan mainstream media, the AKP used two main strategies.

The first strategy was confiscation followed by a manipulated tender. This scenario first took place over the *Star *(a daily newspaper) and *Kanal 24* (a news channel), which had belonged to the Uzan group before the 2001 crisis. In 2004 the TMSF confiscated different media outlets belonging to the Uzan group, including *Star* and *Kanal 24* (cf. Hürriyet 2004, February 14). In 2007, both outlets were sold to a businessman through a public tender organized by TMSF. However, there was another change of ownership of these outlets and they were sold to the AKP-friendly Sancak Holding group (cf. T24 2009, May 20). The second scene was played out in the same year. This time the TMSF confiscated from Ciner Group *ATV* and the *Sabah* newspaper, two of the biggest mainstream outlets at the time, on the grounds that Ciner and its previous owner, the Bilgin group, had signed a secret protocol. The timing of this decision was very meaningful because it was less than 4 months before the 2007 general elections. Neither *ATV* or *Sabah* had a critical position but they were not under the full control of the government and their ratings and circulation were high. So, it was advantageous for the government to silence two important mainstream news outlets for 4 months before the elections. The AKP managed to gain the majority in the 2007 elections once again. After the elections, TMSF organized a public tender, and the only company to make an offer was Çalık Holdings the then chief executive officer of which was Beraat Albayrak, Erdoğan’s son-in-law (cf. Diken 2014b, February 13). For the third act of the confiscation method, in 2013, TMSF confiscated different companies belonging to the Çukurova group, including *Digitürk, Show TV, SkyTürk 360,* and *Akşam*. In the same year, *Sky Türk, Akşam, Güneş* and some other radio channels and magazines were also sold to Sancak Holding.

The AKP’s second strategy for changing the profile of media ownership is to apply tax mobbing and to force them to avoid the media market. The most obvious example was that of the Doğan media, which had controlled more than half of the domestic non-state media market since the mid-1990s. The problems between the Doğan group and the AKP started with the trial of Deniz Feneri launched in Germany. The allegations against the Islamist Deniz Feneri charity were that part of the money it collected was used for other purposes. The indictment cited some members of the AKP, including the then prime minister Erdoğan. When the Doğan media carried the trial on their first page, Erdoğan attacked the Doğan media and blamed media patrons for acting like prosecutors and the courts (cf. DW 2008, September 13). Then, suddenly, the ministry of finance decided to audit Doğan’s financial records for the previous three years (2005, 2006, and 2007) and fined them first $500 million and later $2.5 billion for tax irregularities found during the audit (cf. Arsu and Tavernise 2009, September 9). The first consequence of these fines was that the *Gözcü* newspaper, known to be critical of the AKP, had to shut down, and some critical voices in the mainstream papers *Hürriyet *and *Radikal* were dismissed. Soon afterwards, two important newspapers owned by the Doğan group were sold to Karacan and Demirören Holdings (*Bloomberg HT* 2011, April 20). Although the Doğan group started to decrease its share in the media sector and reduced the level of anti-government criticism, it faced a tacit threat through a continuing criminal case “on charges of ‘establishing an organization for the purpose of criminal activity’, forging official documents and violating Turkey’s anti-smuggling law” (*IPI* 2016, July 8). Finally, in 2018, the Doğan family decided to leave the media and to sell all its media outlets, including *Hürriyet, Kanal D,* and *CNN Türk*, to a conglomerate whose majority shareholder is Erdoğan Demirören, a businessman with close ties to Erdoğan (cf. Bucak 2018, May 29).

In brief, in the last 13 years, using these two strategies the AKP government almost completely changed the ownership profile of the mainstream media. Today, 90% of the mainstream media in Turkey is under the direct control of the AKP government, or more precisely, of President Erdoğan.

### Capture through financial sanctions

The AKP government has also used financial sanctions to discipline those media outlets that they cannot completely capture. The first of these methods targets the advertisement revenues of media companies. This kind of sanction does not often immediately attract attention, but it is very effective. Since most media outlets depend on the advertising revenue, advertising remains a control tactic for governments, especially in non-democratic regimes. The media outlets in Turkey have two sources of advertising revenues: from public announcements and advertisements and from private advertisements. The first group is distributed by the state-run Press Bulletin Authority (Basın İlan Kurumu; BİK), which is controlled by the government. BİK tends to use official adverts and announcements as carrots or sticks, depending on the position of the newspapers and their owners’ relations with the government. According to data compiled by Yanatma, in two years the value of official advertisements given to *Akşam* newspaper increased from TL 5.6 million (in 2013) to TL8 million (in 2015) after the TMSF took control of this newspaper and sold it to a businessman loyal to President Erdoğan. In the same period, due to the confrontation between the AKP and the Gülen movement, official adverts and announcements given to the *Zaman* newspaper (the flagship newspaper of the Gülen movement) decreased from TL 13.5 million to TL5 million (cf. Yanatma 2016, p. 56). A recent example of dropping all official adverts and announcements given to *Evrensel* and *BirGün*, two newspapers known as being leftist and critical of the government, illustrates how BİK can also act as a punitive institution (cf. Köylü 2020, February 5). Given the fact that official adverts constitute important items of revenue, especially for small national newspapers, we can argue that the government aims to silence even miniscule alternative voices using the BİK.

The second group, private advertisement revenues, are also not free from governmental pressure. In his very detailed report, Yanatma studies data showing advertising space (in square centimeters) and analyzes the distribution of advertising in six major public firms (the state-run banks Halkbank, Ziraat Bankası, and Vakıfbank; Turkish Airlines, Turkcell, and Turk Telekom). After a quantitative analysis of advertisement distribution, the author concludes “that the only criterion for public firms advertising distribution is their coverage of the government and circulation did not play any role in this allocation. While these apparently partisan and unfair practices were apparent before 2013 they subsequently turned into a more direct way to punish or support individual newspapers.” (Yanatma 2016, p. 36) During my field research, one of the interviewees who works as a manager at a private TV channel^2^ also referred to an “advertising embargo” by public firms. They argued that once the Minister of Treasury and Finance Berat Albayrak and some partisan columnist known to have close relations to Erdoğan directly criticized and targeted them, an embargo followed. They noted that the negative or critical speeches by high-level AKP members also spread the message to other firms. They added that private firms wanting to keep up good relations with the government stop adverting in these media and this kind of advertising embargo by public firms and companies has an indirect effect on the total of advertisement revenue acquired.

The other method of financial sanctions is more direct and is applied by the Radio and Television Supreme Council (RTÜK).^3^ RTÜK can impose broadcasting bans or heavy penalties to broadcasters for reasons only vaguely defined by law and this can easily become a powerful stick in government hands. Recent experiences show that the government, or more often Erdoğan himself, did not hesitate to use RTÜK as a stick against any critical voice. In response to a parliamentary question from the main opposition party CHP (Cumhuriyet Halk Partisi—Republican People’s Party), RTÜK revealed that it had imposed more than 16,000 sanctions on the media and TL250 million (nearly $45 million) in fines over 8 years from 2010 to 2018 (cf. Güneysu 2019, March 30). The reasons given for these fines include what was defined by the Turkey’s top media watchdog as “targeting, insulting, and threatening Turkish President Erdoğan, in addition to calling for a military coup against the constitution” (cf. IPA 2018, December 27) or critical reporting on the government’s efforts to combat the Covid-19 crisis (*Hürriyet *2020, April 15). Whatever the content, as underlined by Erol Önderoğlu, the representative of *RSF *in Turkey, it is true that alternative media outlets that reject the official discourse are exposed to broadcasting bans and heavy fines (cf. Hacaloğlu 2020, April 17). Additionally, for the first time in the history of RTÜK, media ombudsperson Faruk Bildirici, selected from the CHP quota, was dropped from the council since he criticized almost all the decisions it made and made his criticisms public via social media (cf. Bianet 2019). This exemplifies that the council is reluctant to debate its fines and sanctions and is intolerant to opposition.

### Capture by intimidating and criminalizing journalists

Media patrons or institutions have not been the only target of the AKP. At the same time, it developed a three-step strategy of intimidating journalists trying to do their job. During their first term in office, before full control by ownership of the media had not yet been achieved, members of the AKP government or their representatives made direct contact with the chief editors of TV channels and newspapers. Daily newsroom meetings increasingly took into account their instructions. One of my interviewees^4^ who was working in one of the most important news channels at this period noted that the instructions on which news items to cover started very early. One example was the Pamukova train accident in 2004 where journalists were permitted to cover the tragedy only from limited perspectives and they could not ask questions about the responsibility of the ministry and high-level bureaucrats. The more the AKP reinforced its position within the state bureaucracy over consecutive election victories, the more direct and effective these instructions became. Journalists and correspondents who had good relations with the prime minister were appointed to the positions of chief editor and helped to establish a “red phone line” between the newsroom and the office of the prime minister (cf. Dağıstanlı 2014, p. 102). For example, Nermin Yurteri, who had been the correspondent following Erdoğan, was appointed as the news coordinator of NTV (cf. T24 2011, December 15) and Fatih Saraç^5^ became a member of the management board of the Ciner media group (cf. T24 2014, February 6). Another interviewee^6^ said that there were rumors about WhatsApp groups among new directors of different newspapers and TV channels set by representatives of the prime ministry or presidency. It is also claimed that government representatives can share a complete news article ready to be “copied and pasted in next day’s paper” on these WhatsApp groups (Özer 2018).

The second step for controlling newsrooms was to dismiss journalists who resisted instructions from their chiefs or coming directly from the government. It is difficult to reveal the real number of journalists fired from newsrooms for their critical points of views or refusal to apply instructions dictated by the government. A list of sacked journalists, editors and correspondents prepared by Turkey’s Journalists Union during the Gezi protests alone includes seventy-two names (cf. Muhalefet 2013, July 22). The visible part of this iceberg includes famous columnists, chief editors or anchor persons, such as Banu Güven, Hasan Cemal, Ayşenur Arslan, and Ruşen Çakır (cf. Arsan 2013), who resigned or were dismissed as they could no longer defend the essence and the ethics of journalism.

Although the mainstream media have been captured by the government, step by step, “alternative” or “critical” newspapers or TV channels try to survive against legal and financial sanctions. The third step of intimidation by the government has targeted journalists working in these niche^7^ newsrooms. The AKP’s strategy has been based on the illiberal clauses of the Press Law, the Penal Code and the Anti-Terror Law, which can be very broadly applied according to the desire of the judiciary. The first large-scale judicial attack on journalists started with the Ergenekon case. This began as an investigation into a “deep state” military-led plot to overthrow the AKP government, but soon was transformed into a witch-hunt targeting adversarial voices in media and non-governmental organizations (cf. Steinvorth 2013, August 12). In this period, journalists came up against two categories of charges. Firstly, an important group of journalists who were following the Ergenekon trials were charged, based on the Articles 285 and 288 of the Penal Code, with “the violating of the secrecy of an ongoing trial”. A second group of journalists including famous names such as Mustafa Balbay, Tuncay Özkan, Soner Yalçın, Ahmet Şık, and Nedim Şener was accused of colluding with the so-called Ergenekon ultra-nationalist conspiracy and was jailed pending trial (cf. Yeşil 2016, pp. 99–100). As underlined by one of the interviewees^8^, this harsh judicial suppression orchestrated by AKP’s proxies in the judiciary constituted a breaking point and led to the criminalization of all journalists who do not toe the line. After this moment, journalists who speak truth to power realized in concrete terms that not only their job, but also their freedom was at risk.

After the Ergenekon case, in 2011, 36 journalists working for pro-Kurdish media outlets, namely the *Dicle* news agency and the daily *Özgür Gündem*, were arrested and journalists were charged with membership of KCK (Koma Civaken Kurdistan—Kurdistan Democratic Communities Union) and the PKK, within the framework of police operations that continued for 6 months and targeted administrative staff from the Peace and Democracy Party (BDP), then some academics, and then a large group of lawyers (cf. Ergin 2011, December 28). Journalists were charged with the dissemination of terrorist propaganda and criminalized on the basis of the vague language of the Anti-Terror Law provisions.

The criminalization of journalists gained unprecedented momentum after the failed coup d’état in 2016. A series of judicial suppression targeted journalists with different backgrounds. The first targets were journalists working in media outlets financed by the Gülen movement. Ten days after the coup, arrest warrants were issued for 89 journalists suspected of links with the Gülen movement, some of whom were immediately taken in police custody. More tragically, they found it difficult to get lawyers to defend them (cf. RSF 2016, July 28). On July 28, 2016, all media outlets and print houses close to the Gülen movement were closed down and their movable property as well as all real estate belonging to them were transferred to the Treasury in terms of Decree No. 668. Almost two years afterwards a verdict was handed down and “six former columnists for *Zaman* were sentenced to heavy prison terms for ‘membership in a terrorist organization’” (RSF 2018, July 6).

The second target was the pro-Kurdish newspaper *Özgür Gündem*. The suppression of the newspaper’s editors and journalists was so high that human rights defenders, academics, and journalists initiated a campaign on May 3, 2016 (the day of the Freedom of the Press) to protect their colleagues. Each of the 56 supporters of the campaign became for one day the substitute editor-in-chief of *Özgür Gündem* until August 9. However, the judiciary decided to close down the newspaper temporarily on August 16, 2016 for “regularly making PKK terrorist organization propaganda and serving as a broadcasting organ for an armed terrorist organization” (Akgül 2016, August 16). Next day, some journalists and workers at the daily were detained during a police raid. In addition, the prosecutor charged 38 participants in the solidarity campaign with *Özgür Gündem *with “terror organization propaganda” under the Anti-Terror Act (TMK) Article 7/2 and “releasing or publishing terror organizations’ statements or declarations” under Article 6 (Akgül 2017a, March 7) and their trial started on September 20, 2016 (cf. Duran 2016, September 20). Two different trials related to *Özgür Gündem* lasted for more than 3 years. In brief, the process of jailing and trying defendants in the *Özgür Gündem* trials, as well as the penalties imposed on them, illustrates very well what journalists who do not accept the journalism framework imposed by the government can expect.

The third target was *Cumhuriyet*, one of the oldest newspapers from Turkey’s Republican era. On October 31, 2016 Turkish police detained board members, executives and some columnists following a series of raids (cf. Cumhuriyet 2016, October 31). Nine months after their detention the first hearing of the *Cumhuriyet* trials took place on July 24, 2017. The prosecution claimed that the daily had published news and articles legitimizing the July 2016 coup attempt and given their support to three terrorist organizations—“FETÖ, PKK/KCK and DHKP/C” (a small far-left group) (cf. Akgül 2017b, July 24). The indictment presented all the news and articles criticizing the authoritarian policies of the government as support for terrorism and the court sentenced 13 journalists to prison on terrorism charges on April 25, 2018 (cf. Gall 2018, April 25). As in previous cases against different journalists, the indictment, pretrial detention and trial proceedings of *Cumhuriyet* violated the basic human rights of the defendants. However, the symbolic nature of this trial was even more devastating, as the daily has always had an important place in Turkey’s journalistic history. In addition, one of my interviewees (see footnote 8) noted that the *Cumhuriyet* trials showed that, starting from the Ergenekon case, the AKP government was able to create a very insecure environment and intimidate an important proportion of journalists as well as human rights defenders by using judicial coercion. They explained that it was relatively easier to organize resistance when Ahmet Şık was first arrested during the Ergenekon case. However, they said that everybody was more reluctant to participate when they again tried to organize a campaign to support Ahmet Şık and other *Cumhuriyet* journalists on trial for the second time.

Apart from these major trials, there are several legal investigations against individual journalists. Since the failed coup d’état, the “famous” Article 301^9^ of the Turkish penal code has been put into use again against critical intellectuals including academics, lawyers, and journalists. News covering Turkey’s incursion into Afrin in January 2018 and military operations in Syria were regarded as insults to the Turkish state (cf. Cengiz 2019, October 28). Since 2017, the Media Monitoring Database has registered 36 judicial interferences based on the Article 301 (cf. Medya Gözlem Veritabanı 2020). Turkey’s renewed use of Article 301 as a weapon points out the increasing suppression on journalists.

Additionally, in their report published at the end of 2019, the Committee to Protect Journalists ranked Turkey the second-highest jailer of journalists in the world after China (cf. Beiser 2019). Pretrial detention can last for months and sometimes years and has been used as a method of punishment. In 2017, several cases were brought by journalists to the ECtHR and in March 2018, the Court took an important decision. For the application of Şahin Alpay and Mehmet Altan, the Court held that there had been a violation of Article 5 § 1 (right to liberty and security) and a violation of Article 10 (freedom of expression) of the European Convention on Human Rights (ECHR) and ordered to release these journalists from prison. However, almost immediately another warrant was issued for their arrest on charges and they were sent back to jail (cf. IPI 2020). The last developments show that ECtHR rulings are no longer effectively applied by Turkish government. The fact that Turkey is still a member of the Council of Europe despite the systematic violations of the ECHR ruling creates serious doubts on the capacity of European democratic institutions to deal with new authoritarian regimes such as Turkey.

On the other hand, the huge numbers of prosecutions after the coup attempt in 2016, the use of pretrial detention and the criminalization of any critical views under the state of emergency have increased the number of detainees in prisons and created a very unhygienic and unsafe environment in jail, especially at the time of the global pandemic. Although the Turkish government passed a new prison release law on April 13, 2020, the law fails to include many “political” prisoners who are already convicted or in pretrial detention, including journalists, lawyers, and political and human rights activists. The insistence of the Turkish government on not releasing them brings to mind the question whether it is a conscious choice to leave all political opponents in jails and at a high-level of risk during the Covid-19 pandemic.

## Conclusion

The AKP government has applied threefold strategies to capture the mass media in Turkey. Firstly, they have systematically obliged owners of mainstream media outlets to exit the media sector by using the competences of divers public institutions and imposing financial sanctions. In this way, they have changed the ownership profile of the mass media and created their own loyal and partisan media. Secondly, using RTÜK they have fined media outlets that they cannot fully control. Thirdly, they have targeted and intimidated critical journalists using legal suppression and threats of imprisonment. Different factors, such as the transformation of the political economy after the military coup d’état in 1980, the corrupted relationship between journalists and political leaders, and latent censorship on taboo topics, certainly played a role in facilitating the application of these strategies.

However, the AKP’s methods differed on at least three points from previous governments’ strategies. First of all, they were able to design their media capture strategy within a political system that they have dominated step by step due to their majority in successive elections. Constitutional amendments and important changes in the judicial system have also eroded judicial independence. This erosion of judicial independence opened the way to the criminalization of journalists as well as to intensifying the effects of criminalization.

Secondly, instead of continuing with a stick and carrots strategy with existing media tycoons, they transformed pro-AKP businessmen into media owners. This process created new media patrons whose existence and future depends largely on Erdoğan’s consent. Their blind allegiance to Erdoğan has become the main characteristic of these new media patrons. The transformation of the ownership profile also influenced workers of media outlets at all level. As underlined by one of my interviewees (see footnote 4), an important feature of the journalistic profession is based on a master-apprentice relationship. Yet, the coin of the realm is no longer experience in the field, but the capacity to submit. Those who were able to offer their experience have voluntarily or involuntarily left the profession. The new generation of reporters, editors or columnists is familiar only with the rule of the AKP, which raised important questions about the future.

Thirdly, ever since his very first years in government Erdoğan himself has openly attacked journalists and media patrons with whom he does not have a deal. His threats, “you will pay for this,” directed to critical journalists and the subsequent judiciary suppression that follows these threats refer also to a systemic transformation of the field. This systemic transformation points out an *accreditation of journalism* as a whole in Turkey. In brief, this captured mass media environment in Turkey deeply harms freedom of the press as well as citizens’ right to be informed. However, we are at the same time living in an age of digitalization of the media landscape. Can social media and online news platforms play a role in solving the media freedom problems in Turkey? Or will and can Turkish government apply a digital censorship through the “new social media law regulation”^10^? Further research focusing on the relevance of social media for the freedom of expression and on the impact of social media regulation will permit us to think about media capture through digital censorship.

## Footnotes

1 The Gülen movement is a transnational Islamic order named after the US-based Islamic cleric Fethullah Gülen. Established at the beginning of the 1970s in İzmir, Turkey, the movement became gradually involved in education, politics and the bureaucracy in Turkey (cf. Şık 2017). The partnership or strategic alliance between the AKP and the Gülen movement was obvious from 2002 to 2010. After a hidden confrontation between them from 2010 to 2013, AKP leaders, especially Erdoğan, started to attack the Gülenists (cf. Taş 2018, pp. 397–401). Finally, after the failed coup d’état, the AKP declared the Gülen movement a “Fetullahist terrorist organization/parallel state organization”.

2 Based on an interview conducted by the author on October 11, 2019 in Istanbul.

3 RTÜK is composed of nine members who are elected by the Turkish Parliament from nominees on the basis of the number of MPs of each political party. As the appointment process of its members is not transparent, the council is often criticized for its lack of independence (cf. Media Ownership Monitor 2019).

4 Based on an interview conducted by the author on October 31, 2018 in Istanbul.

5 Fatih Saraç is an Islamist businessman who had close relations with Erdoğan (cf. Diken 2014a, February 7).

6 Based on an interview conducted by the author on October 29, 2018 in Istanbul.

7 Based on an interview conducted by the author on April 26, 2019 in Istanbul.

8 Based on an interview conducted by the author on April 24, 2018 in Istanbul.

9 The original version of the Article 301 accepted in 2005 as a part of the new penal code made it a crime to denigrate “Turkisshness” and Turkish government institutions. Prosecutors used the Article 301 against novelists, academics and journalists including well-known names such as Orhan Pamuk, Hrant Dink (murdered by a Turkish nationalist during the period of his trial), Taner Akçam, and Elif Şafak. In 2008, a new amendment changed “Turkishness” into “the Turkish nation” and made it obligatory to get approval of the minister of justice to file a case. Taner Akçam brought a case against Turkey and the EctHR (European Court of Human Rights) found Turkey guilty of violating freedom of expression (cf. Akçam v. Turkey 2012).

10 The complete name of this regulation accepted on July 28, 2020 by the Turkish Parliament is “Law on the Arrangement of Internet Publication and Combating Crimes Committed Through These Publications”.
